# Effects of Oral Glucosamine Hydrochloride Administration on Plasma Free Amino Acid Concentrations in Dogs

**DOI:** 10.3390/md9050712

**Published:** 2011-04-27

**Authors:** Kazuo Azuma, Tomohiro Osaki, Takeshi Tsuka, Tomohiro Imagawa, Yoshiharu Okamoto, Yoshimori Takamori, Saburo Minami

**Affiliations:** 1 Department of Veterinary Clinical Medicine, School of Veterinary Medicine, Tottori University, 4-101 Koyama-minami, Tottori 680-8553, Japan; E-Mails: kazuazu85@yahoo.co.jp (K.A.); tosaki@muses.tottori-u.ac.jp (T.O.); tsuka@muses.tottori-u.ac.jp (T.T.); imagawat@muses.tottori-u.ac.jp (T.I.); yokamoto@muses.tottori-u.ac.jp (Y.O.); 2 Koyo Chemical Co. Ltd., 2-11-1 Koraku, Bunkyoku, Tokyo 112-0004, Japan

**Keywords:** glucosamine hydrochloride, *N*-acetyl-d-glucosamine, amino acid, dog

## Abstract

We examined the effects of oral glucosamine hydrochloride (GlcN), *N*-acetyl-d-glucosamine (GlcNAc) and d-glucose (Glc) administration on plasma total free amino acid (PFAA) concentrations in dogs. The PFAA concentrations increased in the control group and the GlcNAc group at one hour after feeding, and each amino acid concentration increased. On the other hand, in the GlcN group and the Glc group PFAA concentrations decreased at one hour after feeding. A significant decrease in amino acid concentration was observed for glutamate, glycine and alanine. Our results suggest the existence of differences in PFAA dynamics after oral administration of GlcN and GlcNAc in dogs.

## 1. Introduction

Glucosamine hydrochloride (GlcN) and *N*-acetyl-d-glucosamine (GlcNAc) are components of glycosaminoglycan now widely used as dietary supplements [1]. Moreover, GlcN is useful for the treatment of joint diseases both in humans and in veterinary medicine, including dogs and horses [2,3]. The bioavailability of GlcN has been reported as 26% in humans [4], 19% in rats [5], 12% in dogs [6], and 2–6.1% in horses [7–9]. These results suggest the presence of a species-specific difference in GlcN absorption and metabolism. Different biological activities between GlcN and GlcNAc have also been demonstrated *in vitro*. For example, differences in GlcN and GlcNAc uptake and their subsequent effects on glucose transport, glucose transporter (GLUT) expression, and sulfated glycosaminoglycans (sGAG) and hyaluronan synthesis have been reported [10].

In an experimental rabbit model of cartilage injury, oral administration of GlcN or GlcNAc led to regeneration of both glycosaminoglycan and proteoglycan [11,12]. GlcN has the potential to exert chondroprotective action on an experimentally induced osteoarthritis by inhibiting type II collagen degradation and enhancing type II collagen synthesis in the articular cartilage [13]. These results suggested that synthesis of type II collagen and proteoglycan core protein as well as glycosaminoglycan occurs upon GlcN and GlcNAc supplementation. However, no reports to date have investigated the relationship between oral administration of amino monosaccharide and amino acid synthesis.

The aim of this study was to examine the effects of oral GlcN and GlcNAc administration on plasma free amino acid (PFAA) concentrations. Using dogs, we investigated (PFAA) dynamics after oral administration of GlcN, GlcNAc, or glucose (Glc), which are the sources of glycosaminoglycan and proteoglycan in the body.

## 2. Results and Discussion

**Figure 1.**
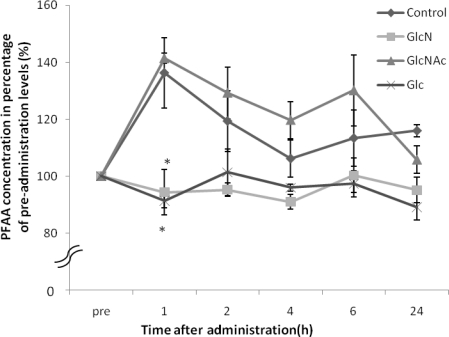
Changes in plasma total free amino acid (PFAA) concentration in dogs after each saccharide (GlcN, GlcNAc, and Glc) administration. Plasma total free amino acid concentration pre-administration was considered as 100%. *: p < 0.05, compared to the level of the control at each hour. Data represent the mean ± SE of three dogs in each group.

Amino acids measured in this study are shown in Table 1. The PFAA concentrations increased in the control dogs and the GlcNAc treated dogs whereas those from the GlcN- or the Glc-treated dogs significantly decreased after one hour (Figure 1). The levels of Glu, Gly, and Ala concentrations were significantly lower than observed fort he GlcNAc-treated dogs (Table 2).

After administration of GlcNAc, no remarkable change was observed in either PFAA concentration or each amino acid level compared to the control.

**Figure 2.**
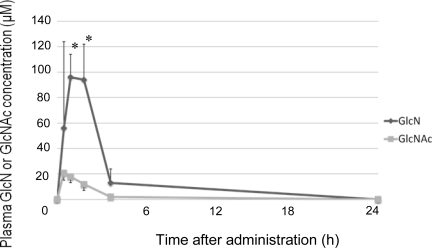
Changes in plasma GlcN or GlcNAc concentration after oral GlcN or GlcNAc administration. All data indicate mean ± S.D. *: P < 0.05 compared to GlcN group.

In a healthy human report, postprandial PFAA concentrations were raised compared to those before a meal [14]. Following feeding, PFAA increased in the control dogs and the GlcNAc-treated dogs. However, PFAA decreased compared to the control group after administration of GlcN or Glc. In an *in vitro* study using mesenchymal stem cells, treatment with 100 μM or 1,000 μM GlcN increased expression of aggrecan and type II collagen. Moreover, 100 μM GlcN treatment led to increased sGAG content [15]. In humans, plasma GlcN concentration reached 150–300 μM after oral administration of 20 mg/kg GlcN [16]. In dogs, plasma GlcN concentration was reported to reach 50 μM after oral administration of 125 mg/kg GlcN [6], and reached 100 μM after oral administration of 300 mg/kg GlcN (Figure 2). Although the maximum plasma GlcN concentration achieved after oral administration of 500 mg/kg GlcN has not been reported, a previous study indicated that it exceeds 100 μM. These findings suggested that high levels of GlcN were provided to the tissue by the circulatory system in dogs. Naito K *et al.* described that GlcN has the potential to exert a chondroprotective action on an experimentally induced OA by inhibiting type II collagen degradation and enhancing type II collagen synthesis in the articular cartilage [13]. Therefore, proteoglycan and type II collagen were likely to be synthesized actively in cartilage. Gly, Ala and Glu are the main components of type II collagen [17], and the levels of these amino acids became lower than those of the control and GlcNAc-treated dogs after administration of GlcN or Glc. These results suggest that GlcN or Glc stimulated proteoglycan and type II collagen synthesis in the dogs.

No change in total or individual amino acid concentrations was observed after administration of GlcNAc compared to the control group. The maximum concentration of GlcNAc in dogs reached about 20 μM after 300 mg/kg GlcNAc administration (Figure 2). Therefore, absorption of GlcNAc by the canine gut may be inferior to that of GlcN. However, the mechanisms of GlcNAc absorption are unclear; further investigation into the absorption and metabolism of GlcNAc is necessary.

In our pilot study using a horse, plasma ammonia concentration increased from 2 h after administration of GlcN. The level of PFAA increased compared to pre-administration levels (data not shown). The presence of a species-specific difference in GlcN absorption and metabolism was suggested [4–9].

We did not directly confirm type II collagen and proteoglycan synthesis in dogs after administration of GlcN. However, oral administration of GlcN induced obvious functional recovery in various kinds of canine orthopedic diseases [2]. To confirm these phenomena occur in dogs is required to understand the mechanism of GlcN in dog joint diseases.

## 3. Experimental Section

### 3.1. Materials

Glucosamine hydrochloride (GlcN) was supplied by Koyo Chemical Co., Ltd., (Tokyo, Japan). *N*-Acetyl-d-glucosamine (GlcNAc) was supplied by Yaizu SuisannKagaku Industry Co., Ltd., (Shizuoka, Japan). d-Glucose (Glc), molecular weight 180.16, was purchased from Wako Pure Chemical (Osaka, Japan).

### 3.2. Animals

Three healthy beagle dogs, mean age of 4 years (range 2–6 years) and mean body weight 9 kg (range 7–12 kg). The use of these animals and the procedures they underwent were approved by the Animal Research Committee of Tottori University.

### 3.3. Administration and Blood Sampling

Dogs were separated into the following groups (n = 3 for each group): usual dog food (Hill’s-Colgate (Japan) Ltd, Science Diet, Tokyo, Japan) (Control), usual dog food plus GlcN, usual dog food plus GlcNAc, and usual dog food plus Glc. Each saccharide dissolved in water was orally administered at approximately 500 mg/kg to dog. Dog’s blood was collected (0 h) in the morning before being fed, then 35 kcal/kg body weight dog food with each saccharide dissolved in 10 mL water (500 mg/kg body weight, single dose) was fed (10 mL water for control group) (first feeding). After blood collection at 1, 2, 4, and 6 h, all the dogs were fed with 35 kcal/kg dog food without saccharide (second feeding), and the blood was collected 18 h after second feeding.

Blood was collected from the jugular vein using heparin as an anti-coagulant. The blood was centrifuged at 3,000 rpm for 10 min, and the plasma was then separated promptly and frozen at −80 °C until measurement of PFAA concentrations.

### 3.4. Measurement of PFAA Concentrations

Plasma samples were mixed with equal volumes of 3% (w/w) sulfosalicylic acid, and left to stand at 4 °C for 1 h. Samples were then centrifuged (4 °C, 15 min, 1,500 rpm), and precipitated protein was removed. The amino acid concentrations were measured by an automatic amino acid analyzer (JLC-500/V2, AminoTac; JEOL, Tokyo, Japan). The amino acids measured are listed in Table 1.

### 3.5. Measurement of Plasma GlcN and GlcNAc Concentrations

GlcN or GlcNAc dissolved in water was orally administered at approximately 300 mg/kg to dogs (n = 3 in each group). Blood samples were collected before administration and 0.5, 1, 2, 4, and 24 h after administration. Blood was collected from the jugular vein using heparin as an anti-coagulant. The blood was centrifuged at 3,000 rpm for 10 minutes, and the plasma was then separated promptly. Plasma samples were mixed with four equal volumes of ethanol and centrifuged, and precipitated protein was removed. These samples were treated using a *p*-ethyl 4-aminobenzoate carbohydrate chain labeling kit (Seikagaku Kogyo, Tokyo, Japan). Samples were analyzed quantitatively using high performance liquid chromatography fitted with a reversed-phase column (Honenpak C18, 75 mm × 4.6 mm I.D.) and fluorometer (Ex. 305 nm, Em. 360 nm).

### 3.6. Statistical Analysis

Each amino acid concentration, total amino acid concentration, essential amino acid concentrations and nonessential amino acid concentrations were used for the evaluation. In dogs, total amino acid concentrations were expressed as the percentage of pre-administration values. Student’s t-tests were used to assess differences at each time point. A probability of 5% or less was considered statistically significant.

## 4. Conclusions

In this study, we investigated differences in plasma amino acid dynamics after oral administration of GlcN, GlcNAc and Glc to dogs. Our results indicate that oral administration in dogs of GlcN or Glu, but not GlcNAc, lowers the levels of some free amino acids in plasma. This result indicates that further work is warranted to determine the significance of this finding for the impact of these dietary supplements on amino acid metabolism and utilization.

## Figures and Tables

**Table 1.** Amino acids measured in this study.

**Essential amino acids (EAA)**	**Nonessential amino acids (NEAA)**
Threonine (Thr)	Asparate (Asp)
Valine (Val)	Serine (Ser)
Methionine (Met)	Asparagine (Asn)
Isoleucine (Ile)	Glutamate (Glu)
Leucine (Leu)	Glutamine (Gln)
Phenylalanine (Phe)	Glycine (Gly)
Lysine (Lys)	Alanine (Ala)
Histidine (His)	Tyrosine (Tyr)
Tryptophan (Trp)	Proline (Pro)
Arginine (Arg)	Citrulline (Cit)
	Ornithine (Orn)
	Hydroxyproline (Hypro)

**Table 2.** Significant changes in serum amino acid concentrations at 1 h after administration of each saccharide (GlcN, GlcNAc, and Glc) to dogs.

	**Control**	**GlcN**	**GlcNAc**	**Glc**
Glu	128.3 ± 6.4	98.6 ± 9.0*	184.0 ± 20.7	103.6 ± 10.1*
Gly	126.8 ± 12.3	95.3 ± 5.8*	145.0 ± 6.1	100.9 ± 2.9*
Ala	135.3 ± 21.9	82.8 ± 4.1*	148.3 ± 4.1	85.4 ± 5.6*

Each plasma free amino acid concentration pre-administration was considered as 100%.

*: p < 0.05, compared to the levels of the GlcNAc or the control at 1 h after administration. Data represent the mean ± SE of three dogs in each group. Control dogs were fed only dog food, and the other dogs were fed dog food supplemented with each saccharide.
